# Copy Number Variants in Patients with Severe Oligozoospermia and Sertoli-Cell-Only Syndrome

**DOI:** 10.1371/journal.pone.0019426

**Published:** 2011-04-29

**Authors:** Frank Tüttelmann, Manuela Simoni, Sabine Kliesch, Susanne Ledig, Bernd Dworniczak, Peter Wieacker, Albrecht Röpke

**Affiliations:** 1 Institute of Human Genetics, University of Münster, Münster, Germany; 2 Department of Medicine, Endocrinology, Metabolism and Geriatrics, University of Modena and Reggio Emilia, Modena, Italy; 3 Department of Clinical Andrology, Centre of Reproductive Medicine and Andrology, University of Münster, Münster, Germany; Temasek Life Sciences Laboratory, Singapore

## Abstract

A genetic origin is estimated in 30% of infertile men with the common phenotypes of oligo- or azoospermia, but the pathogenesis of spermatogenic failure remains frequently obscure. To determine the involvement of Copy Number Variants (CNVs) in the origin of male infertility, patients with idiopathic severe oligozoospermia (N = 89), Sertoli-cell-only syndrome (SCOS, N = 37)) and controls with normozoospermia (N = 100) were analysed by array-CGH using the 244A/400K array sets (Agilent Technologies). The mean number of CNVs and the amount of DNA gain/loss were comparable between all groups. Ten recurring CNVs were only found in patients with severe oligozoospermia, three only in SCOS and one CNV in both groups with spermatogenic failure but not in normozoospermic men. Sex-chromosomal, mostly private CNVs were significantly overrepresented in patients with SCOS. CNVs found several times in all groups were analysed in a case-control design and four additional candidate genes and two regions without known genes were associated with SCOS (P<1×10^−3^). In conclusion, by applying array-CGH to study male infertility for the first time, we provide a number of candidate genes possibly causing or being risk factors for the men's spermatogenic failure. The recurring, patient-specific and private, sex-chromosomal CNVs as well as those associated with SCOS are candidates for further, larger case-control and re-sequencing studies.

## Introduction

Infertility, which affects 10–15% of all couples, is attributed to a male (co-)factor in around 50%. Male infertility is mostly caused by spermatogenic failure, clinically noted as oligo- or azoospermia. However, the reasons for the decreased sperm production remain largely unclear: After a full clinical workup around 30% of cases are considered ‘idiopathic infertile’ and an additional 40% have not sufficient/uncertain causes (e.g. varicocele, infections) [1], [2]. On the other hand, it is estimated that about 30% of azoo-/oligozoospermia are caused by chromosomal abnormalities or mutations of genes involved in germ cell production and function [3]. Thus, male infertility of genetic origin supposedly affects about 1 in 40 adult men and can be considered a common, complex disease.

The currently established genetic causes of male infertility comprise abnormalities on all genomic levels from chromosomal abnormalities, in particular Klinefelter syndrome, to Y-chromosomal (azoospermia factor, AZF) deletions, to mutations of the cystic fibrosis transmembrane conductance regulator (*CFTR*, all gene information - name, location, IDs - available as Table S1) gene. However, although routinely performed in clinical workup [4] karyotyping, AZF deletion screening and *CFTR* sequencing elucidate the reason for infertility in only 5% of unselected and around 20% of azoospermic patients [1], [2].

A multitude of up to 1,500 genes are thought to be involved in spermatogenesis of which 300–600 are specifically expressed in the male germline [5]–[7] and are therefore candidates for causing male infertility. In the last 5–10 years, many efforts have been undertaken with research focused on targeted re-sequencing of a number of these candidate genes. Nevertheless, up to date no additional genetic cause is recognised to be administrable in patient care, mostly because results have either not been replicated or might only explain minute effects [8]–[10].

Copy Number Variants (CNVs) have been shown to be an important source of genetic diversity with remarkable differences between individuals and to play a role in complex diseases such as mental retardation, schizophrenia and cancer [11], [12]. Recently, CNVs have also been analysed in premature ovarian failure (POF), XY gonadal dysgenesis and Mayer-Rokitansky-Küster-Hauser syndrome, diseases linked to genital development and function [13]–[15]. To date, CNVs have not been analysed in men with spermatogenic failure and we hypothesised that CNVs cause spermatogenic failure by either of the following mechanisms: An increased number or specific distribution of CNVs could result in defective recombination, meiotic failure and loss of germ cells. CNVs might also affect the activity of individual genes important for spermatogenesis. We therefore performed high-resolution array Comparative Genomic Hybridisation (array-CGH) in groups of well-characterised idiopathic infertile men with oligo- and azoospermia. For comparison, normozoospermic controls were analysed, because the current databases of structural genomic variation do not provide the necessary information when evaluating spermatogenesis and fertility. We wanted to determine a) whether the number and/or pattern in selected men with spermatogenic failure differs from that in men with normal spermatogenesis; b) whether recurrent CNVs can be identified which might harbour genes involved in spermatogenesis; c) associations with reproductive parameters.

## Materials and Methods

### Study population

Caucasian patients of German origin with idiopathic infertility were selected from the clientele of the Department of Clinical Andrology of the Centre of Reproductive Medicine and Andrology, Münster, a tertiary-referral centre, using the Androbase© database [16]. All participants underwent a complete physical examination including ultrasonographic analysis of the scrotal content. Ejaculates were obtained by masturbation after 2–7 days of sexual abstinence and all semen values were determined in accordance with the then current WHO criteria [17]. All men with known clinical (e.g. maldescended testes, varicocele, infections) and genetic (karyotype anomalies, Y-chromosomal deletions) causes of infertility were excluded. Of these men, one-hundred with ≥20×10/ml sperm concentration, ≥40×10^6^ total sperm count, ≥2 ml semen volume, ≥50% of a+b or ≥25% a motility, high percentage of normal forms (≥10%) were selected as control population with normal spermatogenesis according to WHO criteria [17]. Twenty-one (21%) of these men had previously induced a spontaneous pregnancy in the current or a former relationship. The study populations comprised patients with a) severe oligozoospermia (N = 89 with ≤5×10^6^/ml sperm concentration and ≤10×10^6^ total sperm count) and b) azoospermia and complete bilateral Sertoli-cell-only syndrome (SCOS, N = 37). The diagnosis of SCOS was established if only tubules with Sertoli-cells were detected in bilateral and multilocular (at least two sites per testis) testicular biopsies and the attempt of testicular sperm extraction (TESE) was unsuccessful.

### Ethics statement

All participants gave written informed consent for evaluation of their clinical data and genetic analysis of their donated DNA samples according to a protocol approved by the Ethics Committee of the Medical Faculty in Münster and State Medical Board.

### Array-CGH

Genomic DNA was extracted from peripheral blood by standard methods and analysed at first by the commercially available Human Genome CGH Microarray Kits 244A (Agilent Technologies, Santa Clara, California, USA). This array comprises 236,381 60-mer oligonucleotide probes with a median probe spacing of 8.9 Kb. During the course of the study, the higher-resolution 400K microarray with 411,056 oligonucleotides and a median probe spacing of 5.3 Kb became available. We switched to using this array to be able to compare the impact of smaller CNVs detected with the higher-resolution on spermatogenic failure. Finally, 78 control men and 42 with severe oligozoospermia were analysed with the 244A arrays and 22 and 47 with the 400K arrays. All azoospermic patients with SCOS were analysed with the 400K arrays. Each patient's DNA was compared to 10 pooled DNAs (Promega Human Genomic DNA: Male, Cat.-Nr. G1471). Labelling and hybridisation were performed according to the manufacturer's protocol. In brief, 1 µg of patients' DNA and the pooled control DNA were double-digested with *AluI* and *RsaI* (Promega, Madison, Wisconsin, USA) and subsequently labelled with Cy5- and Cy3-dUTP using the Genomic DNA Enzymatic Labeling kit (Agilent Technologies), respectively. After purification of the labelled DNA by filtration (Microcon YM-30, Millipore, Bilerica, Massachusetts, USA), patient and control DNA were pooled and hybridised with 25 µg of human COT DNA (ArrayGrade KREACot DNA, Kreatech, Amsterdam, The Netherlands) for 40 h at 65°C in the hybridisation oven (Agilent Technologies). After post-hybridisation washes, the arrays were scanned using a Microarray Scanner (G2565BA, Agilent Technologies), and the spot intensities were measured by ‘Feature Extraction Software’ (version 10.7, Agilent Technologies). Analyses and visualisation were performed with ‘DNA Analytics’ (version 4.0.81, Agilent Technologies) with the following parameters: aberration algorithm ADM-2, threshold 6.0, fuzzy zero, centralisation and moving average window 1 Mb. Aberrant signals including 4 or more adjacent probes were considered as genomic CNVs. The raw array-data acquired by the Feature Extraction Software is accessible through the Gene Expression Omnibus (GEO, http://www.ncbi.nlm.nih.gov/geo, series record GSE27965) and the abstracted CNVs were submitted to the Database of genomic structural variation (dbVar, http://www.ncbi.nlm.nih.gov/dbvar, study ID nstd53). CNVs found in normozoospermic controls are also available as Table S2. The Database of Genomic Variants (DGV, http://projects.tcag.ca/variation/) was used to compare findings to previously reported studies. Coordinates of CNVs are based on the National Center for Biotechnology Information (NCBI) Human Genome Build 36 (hg18).

### Statistical analysis

Comparisons between patients and controls were carried out using the two-sample t-tests if data were normally distributed (e.g. number of CNVs) and otherwise non-parametric Mann-Whitney test. Frequencies were compared by Fisher's exact test. In principle, *P*-values less than 0.05 were considered statistically significant. To correct for multiple testing, *P*-values were adjusted according to the Bonferroni-Holm procedure, which is less conservative than the standard Bonferroni correction. As semen parameters were not normally distributed, correlations were calculated for log-transformed values. All calculations were performed with Stata/SE (StataCorp LP, version 9.1, College Station, Texas, USA) or GraphPad Prism version 5.00 for Windows (GraphPad Software, San Diego, California, USA).

## Results

### Clinical data

The clinical parameters of the normozoospermic controls and the two study groups with severe oligozoospermia and azoospermia caused by SCOS are presented in Table 1. Testicular volume was lower and LH as well as FSH higher in the two groups with spermatogenic failure. Patients with SCOS also had lower testosterone serum levels. Duration of abstinence and semen volume were comparable between all groups. Sperm concentration and count as well as percentage of sperm with normal morphology and progressive motility were significantly different by selection.

**Table 1:** Comparison of clinical parameters between the study groups (mean ± standard deviation and median, range (min-max) in brackets).

	Normozoospermic controls (N = 100)	Severe oligozoospermia (N = 89)	Sertoli-cell-only syndrome (N = 37)
**Age [y]**	40.1±4.4 (40, 30–59)	38.7±5.8 (39, 23–58)	40.3±5.4 (40, 29–50)
**Testicular volume [ml]**	58±18 (59, 22–111)	41±13 (39, 12–89)**	29±11 (30, 10–56)**
**LH [U/l]**	3.1±1.2 (3.0, 0.7–7.1)	4.5±2.3** (4.0, 1.5–14.1)	7.8±3.2** (6.7, 3.1–15.6)
**FSH [U/l]**	3.7±2.0 (3.1, 1.3–9.4)	7.7±5.4** (5.7, 1.2–24.5)	15.3±4.9** (21.1, 8.5–37.9)
**Testosterone [nmol/l]**	16.4±5.1 (15.9, 6.9–39.0)	15.3±4.9 (15.0, 4.6–31.3)	14.1±5.8 (12.4, 7.6–34.4)**
**Abstinence [d]**	3.9±1.6 (4, 2–7)	3.8±1.3 (4, 2–7)	4.2±1.2 (4, 2–10)
**Sperm concentration [10^6^/ml]**	77.0±47.0 (62.0, 20.0–231.0)	1.4±1.2** (1.1, 0.1–5.0)	0**
**Semen volume [ml]**	3.9±1.5 (3.5, 2.0–9.0)	3.6±1.4 (3.4, 1.5–9.2)	4.1±1.8 (4.0, 1.0–10.0)
**Total sperm count [10^6^]**	287.3±182.1 (269.5, 40.0–1083.5)	4.5±3.3** (3.8, 0.3–10.0)	0**
**a+b Motility [%]**	53.7±5.9 (54, 36–75)	32.4±13.0** (31, 4–60)	-
**Normal morphology [%]**	16.7±5.2 (16, 10–34)	6.0±5.6** (5, 1–24)	-

**significantly different compared to controls (*P*<0.01 calculated by Mann-Whitney test).

### Comparison of CNVs between groups

**Figure 1:**
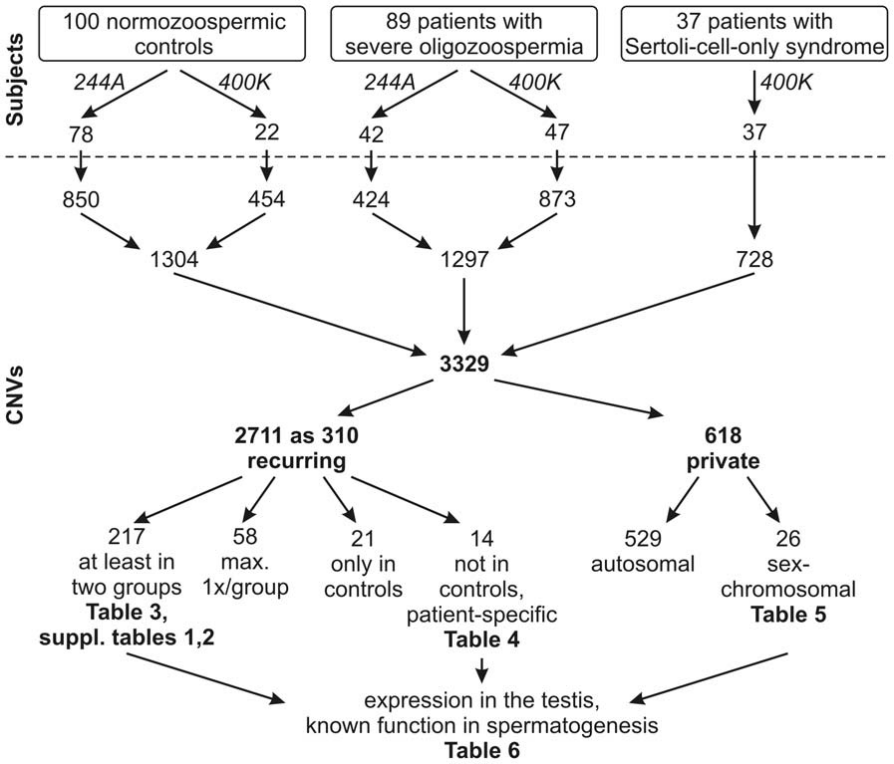
Workflow with different arrays (244A, 400K), number of subjects and categorisation of CNVs.

**Figure 2:**
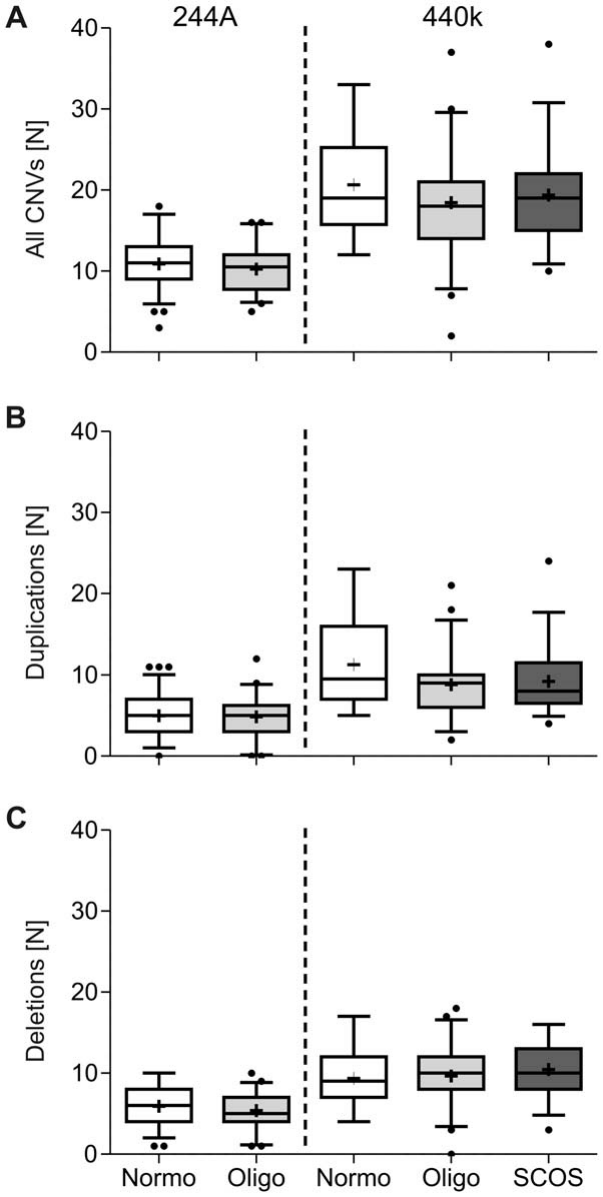
Number of CNVs (A), duplications (B) and deletions (C) separated by array (left: 244A, right: 400K) and patient group (Normo = normozoospermia, Oligo = severe oligozoospermia, SCOS = Sertoli-cell-only syndrome). Around twice as many CNVs (independent of type and fitting the roughly two-fold increased resolution) were detected with the 440K array and in general more duplications than deletions. No significant differences between patient groups were found.

In total, 1304 CNVs were detected in 100 controls, 1297 CNVs in oligozoospermic men (N = 89) and 728 CNVs in patients with SCOS (N = 37); the numbers and categorisations of CNVs are depicted in Figure 1. CNV size ranged from 1.9 Kb to 4.7 Mb with only 5% larger than 1 Mb. The number of detected CNVs and amount of DNA change per men is presented in Table 2 according to study group. As expected from the two-fold increased resolution, around twice as many CNVs and also duplications/deletions separately were found with the higher-resolution 400K array (Fig. 2). The amount of DNA change did not increase likewise with the 400K arrays, as mostly smaller CNVs were additionally detected. Overall, no significant differences in number of CNVs (Fig. 2) or amount of DNA change were found between controls and the two study groups. In addition, particularly large (>500 Kb, >1 Mb) CNVs were analysed, but did not show different distributions between groups.

**Table 2:** Detected CNVs and amount of DNA change per man separately for each array resolution (mean ± standard deviation).

	Normozoospermic controls		Severe oligozoospermia		Sertoli-cell-only syndrome
Array	244A	400K	244A	400K	400K
**CNVs [N]**	10.9±3.0	20.6±5.7	10.2±2.8	18.5±6.3	19.7±5.9
**Duplications [N]**	5.0±2.5	11.3±5.0	4.9±2.4	8.8±3.8	9.2±4.1
**Deletions [N]**	5.9±2.3	9.4±3.0	5.4±2.1	9.6±3.8	10.5±3.4
**DNA change [Kb]**	2732±1335	3076±842	3015±1383	3313±2257	3087±1292
**DNA gain [Kb]**	1510±1234	1649±1144	1842±1414	1761±1219	1464±850
**DNA loss [Kb]**	1223±745	1427±730	1173±932	1551±1841	1624±950

No significant differences were found between the study groups. *P*-values calculated by two-sample t-tests.

**Figure 3:**
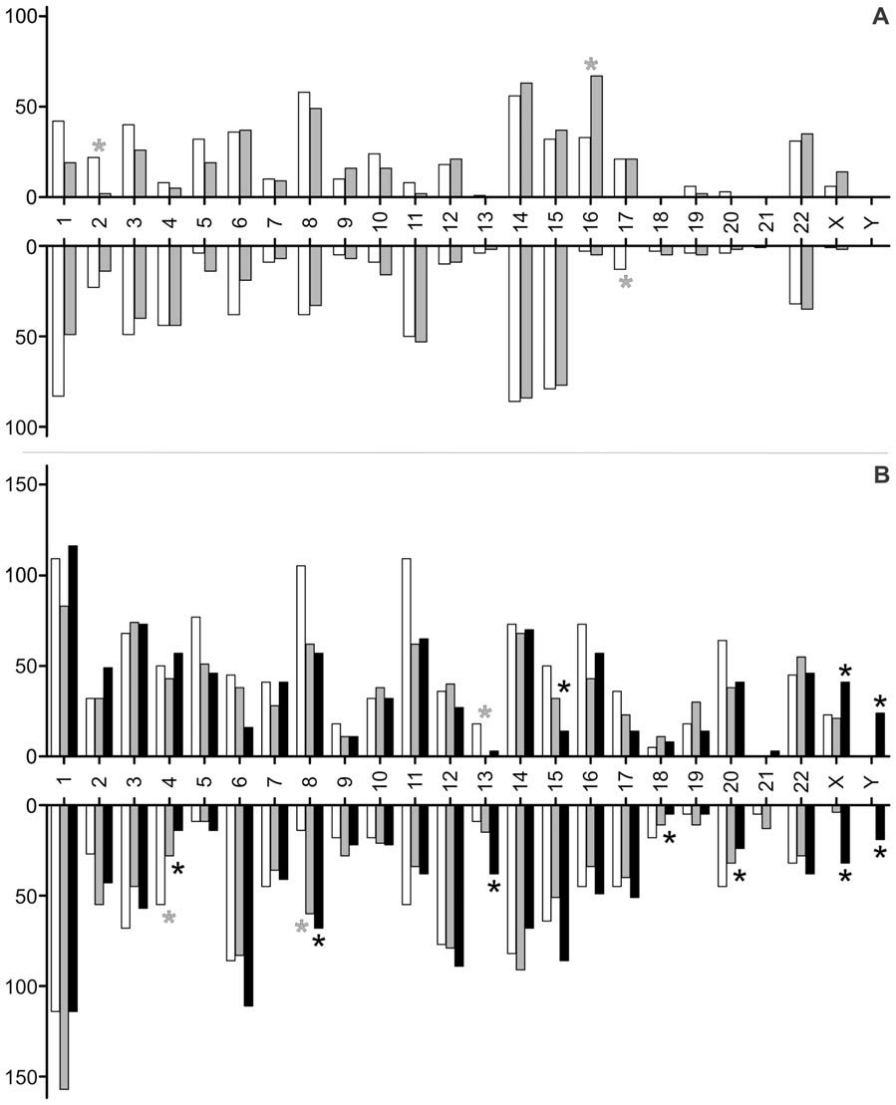
Number of duplications (upwards) and deletions (downwards) per chromosome (normalised per 100 men) detected by 244A (A) and 400K (B) arrays for normozoospermic controls, oligozoospermic and SCOS patients (open, grey and black bars, respectively). Significantly different frequencies between the groups are marked with an asterisk. *P*-values calculated by Fisher's exact test. To correct for multiple testing, *P*-level for significance was adjusted according to the Bonferroni-Holm procedure.

To analyse whether the chromosomal distribution of CNVs was different between the groups, the number of CNVs, and duplications/deletions separately, was calculated per chromosome and normalised to 100 men (Fig. 3). The overall distribution of duplications and deletions was found to be significantly different between all groups (*P*<0.01) and for both types of arrays. The *post-hoc* comparison of number of CNVs per single chromosome indicated significant differences between patients and controls for several chromosomes after Bonferroni-Holm correction. The detailed per-chromosome analysis revealed no specific hotspots associated with oligozoospermia or SCOS on the respective chromosomes. In contrast, the differences in the distribution were caused by recurring CNVs that were found more or less frequently in the respective study groups (see below).

In many cases, several differently sized CNVs spanned a common region but probably have different breakpoints. If either the gene content was identical or the CNVs spanned regions with the breakpoints distance ±10 Kb (roughly corresponding to one oligonucleotide and thereby the minimum array resolution), these CNVs were aggregated for statistical analyses. Duplications and deletions were considered as different variants because of their supposedly diverse impact. Of the 3329 CNVs, a majority of 2711 (81%) could thereby be summarised as 310 recurring variants (159 deletions and 151 duplications), of which the smallest common region is then reported. Some of these were only found in normozoospermic controls (N = 21), at most once per group (N = 58) or not in controls (N = 14, recurring, patient-specific CNVs).

**Figure 4:**
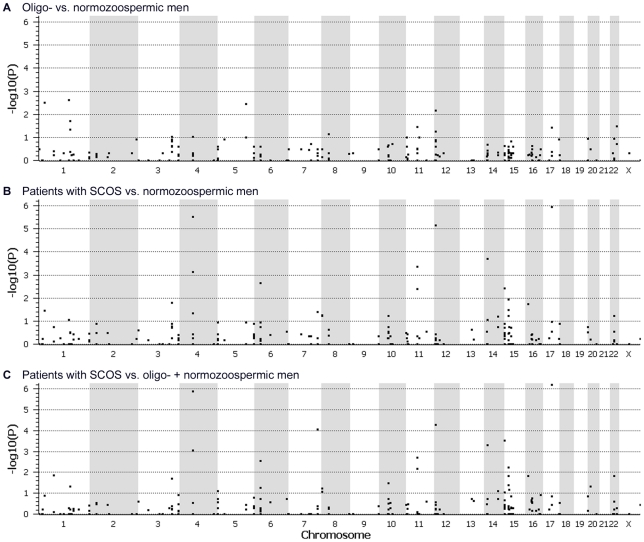
Plots of −log *P*-values of frequency comparisons for all recurring CNVs found in patients and controls grouped by chromosome. *P*-values calculated by Fisher's exact test. The figure does not include the Y chromosome because no recurring variants were found on it.

The frequencies of the remaining 217 variants were compared between the study groups (Table S3 and Table S4). Plots of the −log(10)*P*-values (Manhattan-plots) of case-control comparisons are presented in Fig. 4. To correct for multiple testing, the *P*-value for selection of candidate CNVs was set to 0.001 and these 6 are reported in Table 3. Except the decreasing frequency of a deletion on 4q13.1 including *UGT2B17* and a small duplication on 14q32.33 without known genes, all differences remained significant also after Bonferroni-Holm correction. Ten recurring CNVs were only found in patients with severe oligozoospermia, while three were SCOS specific and one CNV (3p11.1 in an intron of *EPHA3*) was found in both groups with spermatogenic failure but not in normozoospermic men (Table 4). Already from the chromosomal distribution it became clear, that sex-chromosomal CNVs were significantly overrepresented in patients with SCOS. These are all private variants summarised in Table 5. Both groups of patient-specific CNVs (Table 4 and 5) were marked as ‘likely pathogenic’ in dbVar. All genes in either CNVs with significantly different frequencies between patients and controls, patient-specific CNVs or private, sex-chromosomal CNVs (Tables 3, 4, and 5) were checked through PUBMED (http://www.ncbi.nlm.nih.gov/pubmed) and OMIM (http://www.ncbi.nlm.nih.gov/omim) searches. Those genes with known expression in the testis and/or function related to spermatogenesis are presented in Table 6.

**Table 3:** The 6 recurring CNVs with significantly different (P<0.001, marked in bold) frequencies (number of cases/all cases*; percentage in brackets) between patients with oligozoospermia or SCOS and normozoospermia or between SCOS and both other groups combined.

Region	Start	End	Size [Kb]	Gene symbol(s)	Type	Normozoosp.	Sev. oligozoosp.	*P*	Sertoli-cell-only syndrome	*P*	*P* SCOS vs. others	*DGV*
4q13.2	69069451	69166014	96.6	*UGT2B17*	dup	10/100 (10%)	12/89 (13.5%)	0.50132	18/37 (48.6%)	**0.00000**	**0.00000**	yes
4q13.2	69069451	69166014	96.6	*UGT2B17*	del	28/100 (28%)	21/89 (23.6%)	0.51072	1/37 (2.7%)	**0.00072**	**0.00086**	yes
7q34	141413152	141438704	25.6	*MGAM*	dup	1/22 (4.5%)	0/47 (0%)	0.31884	10/37 (27%)	0.04059	**0.00009**	yes
7q34	141413152	141438704	25.6	*MGAM*	del	3/22 (13.6%)	8/47 (17%)	1.00000	4/37 (10.8%)	1.00000	0.56819	yes
12p13.31	9528390	9610254	81.9		dup	1/100 (1%)	9/89 (10.1%)	**0.00683**	0/37 (0%)	1.00000	0.37395	yes
12p13.31	9528390	9610254	81.9		del	4/100 (4%)	11/89 (12.4%)	0.05618	13/37 (35.1%)	**0.00001**	**0.00005**	yes
14q11.2	19268576	19490830	222.3	*OR4Q3*, *OR4M1*, *OR4N2*, *OR4K2*, *OR4K5*, *OR4K1*	dup	10/100 (10%)	13/89 (14.6%)	0.37765	8/37 (21.6%)	0.08990	0.18733	yes
14q11.2	19268576	19490830	222.3	*OR4Q3*, *OR4M1*, *OR4N2*, *OR4K2*, *OR4K5*, *OR4K1*	del	36/100 (36%)	24/89 (27%)	0.21172	2/37 (5.4%)	**0.00020**	**0.00049**	yes
14q32.33	105602402	105630289	27.9		dup	8/100 (8%)	5/89 (5.6%)	0.57633	11/37 (29.7%)	**0.00370**	**0.00029**	yes
14q32.33	105602402	105630289	27.9		del	14/100 (14%)	12/89 (13.5%)	1.00000	7/37 (18.9%)	0.59345	0.44578	yes
17q21.31	41521344	41566740	45.4	*KIAA1267*	dup	10/100 (10%)	11/89 (12.4%)	0.64844	1/37 (2.7%)	0.28787	0.13883	yes
17q21.31	41521344	41566740	45.4	*KIAA1267*	del	6/100 (6%)	9/89 (10.1%)	0.41980	16/37 (43.2%)	**0.00000**	**0.00000**	yes

For comparison, the corresponding duplication/deletion (if present in any group) is included independent of *P*-value. CNVs were checked for occurrence in the Database of Genomic Variants (DGV).

* If CNVs were only found by higher-resolution 400K-array, the number of all cases is reduced to 22 for normozoospermic controls and to 47 for patients with severe oligozoospermia (see methods).

SCOS = Sertoli-cell-only syndrome. *P*-values calculated by Fisher's exact test. To correct for multiple testing, *P*-level for significance was adjusted according to the Bonferroni-Holm procedure. Gene information - name, location, IDs - available as Suppl. Table S1.

**Table 4:** The 11 and 4 recurring, patient-specific CNVs not found in normozoospermic controls with number of cases, type (dup = duplication, del = deletion), gene content and whether the CNV was described in the Database of Genomic Variants (DGV).

Group	Region	Start	End	Size (Kb)	Number, type	Gene symbol(s)	DGV
**Oligozoospermia**	2p11.2	89635198	89902565	267.0	2xdel	-	yes
	3p11.1	89476719	89499633	22.0	4xdel	*EPHA3*	yes
	4p16.1	8235974	8261720	25.7	2xdup	*SH3TC1*	yes
	6p21.31	35143115	35184210	41.1	2xdup	*ANKS1A*	no
	10q23.1	84138134	84171245	33.1	2xdel	*NRG3*	no
	10q23.33	96497202	96536412	39.2	2xdel	*CYP2C19*	no
	12q13.3	55866674	55896055	29.4	2xdup	*LRP1*, *MIR1228*	no
	16q22.1	66942648	66967713	25.1	2xdup	*PRMT7*, *SMPD3*	no
	17q12	30624580	30787596	163.0	2xdel	*SLFN11*, *SLFN12*, *SLFN13*	no
	18q23	75746093	75779459	33.0	1xdup, 2xdel	*KCNG2*, *PQLC1*	no
	Xq26.3	134120502	134157976	37.5	2xdup	*CXorf48*	yes
**SCOS**	3p11.1	89476719	89499633	22.9	3xdel	*EPHA3*	yes
	8q24.3	145061948	145093349	31.4	1xdup, 1xdel	*PLEC*, *MIR661*	yes
	12p11.21	31132516	31223665	91.1	2xdel	*DDX11*, *OVOS2*	yes
	12q23.1	98491661	98519308	27.6	2xdel	*ANKS1B*	no

SCOS = Sertoli-cell-only syndrome. Gene information - name, location, IDs - available as Table S1.

**Table 5:** The 12 and 14 private, sex-chromosomal, patient-specific CNVs not found in normozoospermic controls with type (dup = duplication, del = deletion), gene content and whether the CNV was described in the Database of Genomic Variants (DGV).

Group	Region	Start	End	Size (Kb)	Type	Gene symbol(s)	DGV
**Oligozoospermia**	Xp21.3	28162190	28214748	52.0	dup	-	yes
	Xp11.4	38376283	38513841	137.6	dup	*TSPAN7*	no
	Xp11.22	52657689	52978139	320.5	dup	*SSX7*, *SSX2*, *SPANXN5*, *XAGE5*, *XAGE3*, *FAM156A*, *FAM156B*	yes
	Xp11.22	52842080	52909890	67.8	dup	*SPANXN5*, *XAGE5*, *XAGE3*	no
	Xq22.1	102134796	102496321	361.5	dup	*BEX1*, *NXF3*, *BEX4*, *TCEAL8*, *TCEAL5*, *BEX2*, *TCEAL7*	no
	Xq22.2	103066101	103190187	124.0	dup	*TMSB15B*, *H2BFXP*, *H2BFWT*, *H2BFM*	yes
	Xq22.3	105010614	105561054	550.4	dup	*NRK*, *SERPINA7*, *MUM1L1*	yes
	Xq22.3	110238448	110260226	21.0	dup	*PAK3*	no
	Xq23	111598447	111621531	23.0	del	*-*	no
	Xq25	123911267	124039708	128.4	del	*ODZ1*	no
	Xq27.1	139706586	139904507	197.9	dup	*MIR320D2*	no
	Xq28	154044877	154079019	34.0	del	*-*	no
**SCOS**	Xp22.33	2711073	2814530	103.5	del	*XG*, *GYG2*	no
	Xp22.2	16688233	16707403	19.2	dup	*SYAP1*	no
	Xp21.3	25568263	25583583	15.3	del	*-*	no
	Xp11.3	44067590	44084085	16.5	dup	*EFHC2*	no
	Xq11.1	64806000	64854709	48.7	dup	*MSN*	no
	Xq12	65385501	65413711	28.2	dup	*HEPH*	no
	Xq22.3–q23	110226892	110965127	738.2	dup	*PAK3*, *CAPN6*, *DCX*, *ALG13*, *TRPC5*	no
	Xq24	118780844	118798128	17.3	dup	*-*	no
	Xq25	122920543	123009115	88.6	dup	*STAG2*	no
	Xq26.2	131413847	131439663	25.8	del	*MBNL3*	no
	Xq26.3	134600709	134628136	27.4	dup	*-*	yes
	Yp11.2	7348864	7491480	142.6	dup	*-*	no
	Y11.223	21964794	22058959	94.2	dup	*RBMY2EP (AZFb/bc)*	yes
	Y11.23	26870161	27073218	203.1	dup	-	yes

SCOS = Sertoli-cell-only syndrome. Gene information - name, location, IDs - available as Table S1.

**Table 6:** Candidate genes with proposed function.

Group	Phenotype	Region	Gene symbol	OMIM	Function
**Risk factor**	Normo, Oligoz., SCOS	4q13.2	*UGT2B17*	601903	glucuronidase essential for urinary testosterone excretion [23], [24]
**Recurring, patient-specific CNVs**	4xOligoz./3xSCOS	3p11.1	*EPHA3*	179611	Anks proteins involved in modulating degradation of EphA receptors [25]
	2xOligoz.	6p21.31	*ANKS1A*	608994	
	2xSCOS	12q23.1	*ANKS1B*	607815	
	2xSCOS	8q24.3	*PLEC*	601282	Plectin in Sertoli cells concentrated at intercellular junctions and nuclear surface [29]
	2xOligoz.	16q22.1	*PRMT7*	610087	protein methyltransferase, cooperates with the testis-specific factor CTCFL [30]
**Private, sex-chromosomal, patient-specific CNVs**	1xOligoz.	Xp11.4	*TSPAN7*	300096	interaction with SPAG11B (sperm associated antigen) isoform D, associated with spermatozoa [31]
	1xOligoz.	Xp11.22	*SSX7*	300542	cancer-testis antigen, expressed in normal testis tissue [32], [33]
	1xOligoz.	Xp11.22	*SPANXN5*	300668	cancer-testis antigen, expressed in post-meiotic spermatids [34]
	1xOligoz.	Xq22.1	*BEX1*	300690	in mice: expression in pachytene spermatocytes and spermatids [35]
	1xOligoz.	Xq22.1	*NXF3*	300316	belongs to a family of nuclear RNA export factors (NXF), Nxf2 plays a role in spermatogenesis (meiosis and maintenance) [36], [37]
	1xOligoz.	Xq22.2	*H2BFWT*	300507	testis-specific histone, SNP in 5′ untranslated region associated with oligozoospermia [38]–[40]
	1xOligoz./1xSCOS	Xq22.3	*PAK3*	300142	one isoform specifically expressed in testis [41]
	1xSCOS	Xq23	*TRPC5*	300334	interacts and co-localises with Enkurin in sperm [42]

OMIM = Online Mendelian Inheritance in Man (http://www.ncbi.nlm.nih.gov/omim); Oligoz. = Oligozoospermia; SCOS = Sertoli-cell-only syndrome. Gene information - name, location, IDs - available as Table S1.

### Association of CNVs with semen parameters

**Figure 5:**
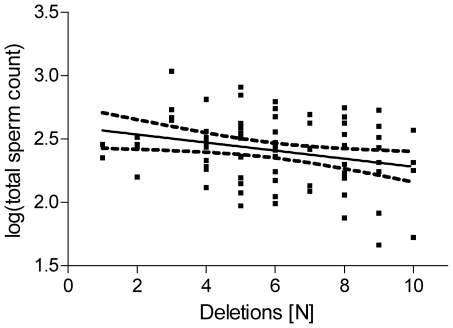
Significant negative correlation (r = −0.270, P<0.05) of total sperm count with number of deletions in 78 normozoospermic men analysed by 244A array. *P*-value calculated on log-transformed total sperm counts.

Under the hypothesis that accumulating CNVs might lead to reduced sperm output, the number of total CNVs, duplications and deletions as well as amount of DNA change was correlated with sperm concentration and count. Because the number of deletions varies strongly with the array used, these were analysed separately. In the largest group, 78 normozoospermic men analysed by 244A arrays, a significant negative association (r = −0.27, *P* = 0.017, Fig. 5) of number of deletions and total sperm count was found. Sperm concentration also showed a trend to be associated with number of deletions (r = −0.22, *P* = 0.055). These correlations were strengthened by corrections for age and abstinence time (*P* = 0.007 and *P* = 0.051) but could not be confirmed in the other, however smaller, groups of men analysed by 400K and/or oligozoospermia. Neither correlations with sperm motility nor morphology were found in any group.

## Discussion

For the first time, we analysed 89 strictly selected patients with severe oligozoospermia, 37 with azoospermia due to SCOS and 100 with normal spermatogenesis as controls by array-CGH. Although interpretation of the results would have been more straightforward if only one type of microarray had been used and while knowing that amount of DNA available as well as funding would not permit us to repeat the analyses on the first set of samples, we favoured switching to the higher resolution array to detect smaller CNVs in almost half of our subjects, which in the eand appeared to be beneficial. As result, we report several genes and genomic regions on autosomes and more pronouncedly on the sex-chromosomes that might either be risk factors (also found in controls) or causative by themselves (not found in controls) for spermatogenic failure.

We hypothesised that an increased number or specific distribution of CNVs could result in defective recombination, meiotic and thereby spermatogenic failure. Structural chromosomal aberrations are found more frequently in men with oligo- and azoospermia with an emphasis on autosomal translocations in the former (3–4% compared to 0.5–1.5% in controls) and sex-chromosomal aneuploidy in the latter (13–16% compared to 0.5–1%) [18]–[20]. The causal relation between chromosomal rearrangements and impaired sperm production has been suggested to be a structural effect related to alterations in the process of chromosome synapsis during meiosis [21], but whether submicroscopic chromosomal rearrangements (CNVs) can result in meiotic recombination defects is not known. By comparing the number of all CNVs, duplications and deletions separately and amount of DNA change, gain and loss no significant differences were found between the groups analysed. The differences in chromosomal distribution of CNVs were attributed to single, recurring CNVs (see below). Also particularly large (>1 Mb) variants were not found more frequently in oligozoospermia or SCOS. Therefore, only even larger variants of several megabases, microscopically detectable upon conventional karyotyping, might impair chromosome synapsis and meiosis. Whether a size threshold for chromosomal aberrations having such a “direct”, gene-independent impact on spermatogenesis exists cannot be concluded from the presented data. It seems, however, that structural variation below the detection limit of routine karyotyping should not be regarded, *per se*, as an obligate cause of spermatogenic failure. Considering that translocation carriers may have normal spermatogenesis [18]–[20], the link between (large) structural chromosomal variation and spermatogenesis remains to be elucidated.

In principle, CNVs may result in altered gene transcription/protein function through different mechanisms: they might encompass dosage-sensitive genes, a deletion may demask a recessive mutation on the homologous chromosome, genes overlapped by structural variation may be disrupted directly or a CNV can exert position effects [22]. By comparing CNVs between the controls and the two patient groups, 11 and 4 CNVs specific for severe oligozoospermia and SCOS, respectively, were identified in more than one patient each (Table 4). In addition, 12 and 14 private, sex-chromosomal CNVs were listed (Table 5) because these may have a functional consequence of the naturally haploid genes contained therein. Also private, autosomal CNVs currently found in one patient each may contribute to the infertility phenotype, but their significance may only be evaluated by much larger studies. According to our moderately conservative approach, six CNVs were found in highly significantly different frequencies in patients with SCOS but also in controls and may be new risk factors associated with male infertility. All of these CNVs remained significant also after Bonferroni-correction. Interestingly, only one CNV in 12p13.31 without known genes was associated with severe oligozoospermia. The 81.9 Kb deletion was found with increasing frequency from controls (4%) to oligozoospermic men (12%) to men with SCOS (35%) as the most severe phenotype. However, the common deletion polymorphism in *UGT2B17* was found in decreasing frequency while a duplication of the same locus was found in increasing frequency. These glucoronidase variants determine urinary excretion of testosterone [23], [24], but have not been studied in male infertility.

Genes may be prioritised according to a known function or indirectly through their expression in the testis. Therefore, the 4 autosomal and 8 X-chromosomal genes presented in Table 6 are the distilled outcome of the current study with respect to identification of new, promising candidate genes. Most of these were shown to be expressed in Sertoli or germ cells, but their functions are mostly unknown. *EPHA3*, *ANKS1A* and *ANKS1B* were mentioned because the Anks proteins were found to modulate degradation of EphA receptors in mice [25] and therefore the three genes might represent members of a functional unity. CNVs altering one of these genes were found in 6 men (7%) with severe oligozoospermia and 5 (14%) with SCOS.

In two recent studies, our group analysed patients with POF and XY gonadal dysgenesis by array-CGH [13], [14]. Interestingly, the putative causal genes identified were enriched for genes also implied in spermatogenesis. When comparing results of these and the current study, CNVs in the *PLEC*, *TSPAN7*, *PAK3*, *TRCP5*, *H2BFWT* loci were found not only in men with SCOS, but also in either patients with POF or XY gonadal dysgenesis (personal communication). These cover 5 of 11 genes identified in the current study which might hint to a common genetic origin of loss of spermatogonia in the male and loss of oogonia in the female resulting in SCOS, XY gonadal dysgenesis and POF, respectively.

With sperm counts ranging from zero to hundreds of millions per ejaculate, sperm output may be viewed as a quantitative trait and male infertility is sometimes postulated as a polygenic disease [26]. Some evidence for this hypothesis may be gained from the significant negative correlation between sperm counts and number of deletions described in our 78 normozoospermic men analysed by 244A array. Especially as hundreds of genes are implicated in spermatogenesis, it is plausible that more deletions also more often involve spermatogenesis-relevant genes. Therefore, a man bearing more deletions may have a less efficient spermatogenesis and therefore lower sperm output. We could, however, not detect a comparable correlation in the, albeit at most half as large, groups of normozoospermic men analysed by 400K arrays or in oligozoospermic patients (irrespective of array used).

While on the one hand the selection of our control group from the patient clientele avoids population stratification, on the other hand the conclusions drawn primarily remain limited to the phenotype of spermatogenic failure and cannot be readily extended to fertility. For this purpose, a group of proven fertile men (fathers) would be needed as additional controls. However, at least one-fifth of our normozoospermic controls had fathered a child before then presenting with either secondary infertility or infertility in a new relationship. Contrariwise, the usually utilised Database of Genomic Variants (DGV) of ‘healthy’ controls is not amenable to be used with respect to the phenotype of spermatogenic impairment, as the fertility status (let alone spermatogenesis) is unknown. Thus, as a larger group of proven fertile men was neither available to us nor has - to our knowledge - been analysed anywhere else yet, our control group may well be used to study spermatogenesis. The CNV data of the 100 controls is provided as supplement as well as accessible through dbVar and will be valuable for other studies on genetics of male infertility.

In contrast to many candidate-gene approaches, only one recently published study analysed 80 men with normozoospermia, 52 with OAT, and 40 with non-obstructive azoospermia (histology not mentioned) using whole genome SNP arrays [27]. Twenty-one SNPs were found significantly associated with azoo- or oligozoospermia, but only four could be replicated in a larger follow-up study [28]. We applied a more stringent patient selection especially for the group of azoospermia as the known histology of SCOS leads to one of the most homogeneous phenotypes possible in male infertility. Accordingly, we could identify a larger number of putative causal CNVs/genes in SCOS than in oligozoospermia. All the recurring, patient-specific and private, sex-chromosomal CNVs as well as the CNVs associated with SCOS described herein are candidates deserving further detailed analyses in larger patient and control groups as well as other populations. Especially with respect to the sex-chromosomal CNVs, analyses of trios would be helpful to determine their relevance. It should be considered, however, that involving the parents of infertile patients is very difficult in comparison to other diseases.

In conclusion, by the first CNV study in male infertility, we provide evidence that CNVs contribute to the complex origin of male infertility and present a number of candidate genes possibly causing or being risk factors for spermatogenic failure.

## Supporting Information

Table S1 **Information about mentioned genes (OMIM = Online Mendelian Inheritance in Man,** http://www.ncbi.nlm.nih.gov/omim **).**
(XLS)

Table S2 **CNVs found in 100 normozoospermic controls.**
(XLS)

Table S3 **All recurring deletions with comparison of frequency (number of cases/all cases; percentage in brackets) between groups.** If CNVs were only found by higher-resolution 400K-array, the number of all cases is reduced to 22 for normozoospermic controls and to 47 for patients with severe oligozoospermia (see methods). *P*-values calculated by Fisher's exact test. SCOS = Sertoli-cell-only syndrome.
(XLS)

Table S4 **All recurring duplications with comparison of frequency (number of cases/all cases; percentage in brackets) between groups.** If CNVs were only found by higher-resolution 400K-array, the number of all cases is reduced to 22 for normozoospermic controls and to 47 for patients with severe oligozoospermia (see methods). *P*-values calculated by Fisher's exact test. SCOS = Sertoli-cell-only syndrome.
(XLS)
